# Short-Beam Shear Strength of New-Generation Glass Fiber-Reinforced Polymer Bars Under Harsh Environment: Experimental Study and Artificial Neural Network Prediction Model

**DOI:** 10.3390/polym16233358

**Published:** 2024-11-29

**Authors:** Mesfer M. Al-Zahrani

**Affiliations:** 1 Civil and Environmental Engineering Department, King Fahd University of Petroleum & Minerals, Dhahran 31261, Saudi Arabia; mesferma@kfupm.edu.sa; 2 Interdisciplinary Research Center for Construction and Building Materials (IRC-CBM), King Fahd University of Petroleum & Minerals, Dhahran 31261, Saudi Arabia

**Keywords:** glass fiber-reinforced polymer (GFRP) bars, durability, accelerated aging, short-beam shear strength, artificial neural networks, linear regression

## Abstract

In this study, the short-beam shear strength (SBSS) retention of two types of glass fiber-reinforced polymer (GFRP) bars—sand-coated (SG) and ribbed (RG)—was subjected to alkaline, acidic, and water conditions for up to 12 months under both high-temperature and ambient laboratory conditions. Comparative assessments were also performed on older-generation sand-coated (SG-O) and ribbed (RG-O1 and RG-O2) GFRP bars exposed to identical conditions. The results demonstrate that the new-generation GFRP bars, SG and RG, exhibited significantly better durability in harsh environments and exhibited SBSS retentions varying from 61 to 100% in SG and 90–98% in RG under the harshest conditions compared to 56–69% in SG-O, 71–80% in RG-O1, and 74–88% in RG-O2. Additionally, predictive models using both artificial neural networks (ANNs) and linear regression were developed to estimate the strength retention. The ANN model, with an *R*^2^ of 0.95, outperformed the linear regression model (*R*^2^ = 0.76), highlighting its greater accuracy and suitability for predicting the SBSS of GFRP bars.

## 1. Introduction

Glass-fiber-reinforced polymer (GFRP) bars are increasingly used in infrastructure projects as a substitute for steel rebars owing to several advantages over traditional steel, including corrosion resistance, a high strength-to-weight ratio, impressive tensile strength, and non-conductive and non-magnetic properties [1,2,3]. Although significant research and field evaluations have resulted in the standardization of GFRP bars as reinforcements in concrete structures [4,5,6], more durability investigations are crucial to understanding their performance in harsh environments, such as alkaline and marine conditions, which can critically impact their long-term service.

Strength degradation in GFRP bars is largely influenced by high temperature and alkaline exposure. The glass transition temperature (*T_g_*) of the polymeric resin matrix, i.e., the point at which the resin softens, weakening the composite bond between glass fibers and the matrix, occurs between 100 and 120 °C [7]. The softening of the polymeric resin results in a viscoelastic transition that causes partially irreversible thermal damage to the matrix. The short-beam shear strength (SBSS), as per ASTM D4475-21 [8], is a vital property for evaluating the quality and performance of GFRP bars as it measures the strength of the bond between the polymeric resin matrix and glass fibers, which directly impacts the composite’s ability to withstand stress without separation. For quality control, SBSS tests provide insights into the consistency of composite materials, enabling manufacturers to identify and rectify variations in production that could lead to premature failures [6,8,9].

Accelerated aging methods, commonly used by researchers [10,11,12,13], are recommended in provisional documents, such as American Concrete Institute’s (ACI) PRC-440.3R-12 [5], to predict the long-term performance of fiber-reinforced polymer (FRP) bars. In these tests, bar specimens are immersed in solutions that simulate the alkaline conditions of Portland cement concrete pore water, typically at 60 °C. By correlating accelerated aging outcomes with real-world exposure effects, these tests provide a basis for estimating the potential strength degradation over time [14]. 

Yu et al. [15] studied the durability performance of GFRP bars in alkaline environments by immersing them in distilled water and simulating concrete pore solutions with pH values of 10 and 13 at temperatures ranging from 21 to 60 °C. The SBSS of the bars was notably affected by higher pH and temperature, with observed strength reductions of approximately 30% at 40 °C and 38% at 60 °C after 100 days of exposure at pH 13. Degradation is primarily due to fiber–matrix interface debonding, particularly at high temperatures. 

Lu et al. investigated the mechanical strength degradation of GFRP bars of 12 mm and 16 mm diameters exposed to elevated temperature levels, focusing on tensile, flexural, and shear properties at temperatures up to 500 °C [16]. The results reveal that the tensile, flexural, and shear strengths showed phased declines across three temperature ranges: 20–120, 120–270, and 270–420 °C. At 270 °C, flexural strength retention was approximately 70%, while at 420 °C, it dropped to approximately 4%, with complete degradation occurring by 500 °C. A simplified predictive model for residual strength was proposed, including a fire-resisting reduction factor (CF) for design, which suggested CF values of 0.65 for tensile strength and 0.75 for other strengths in GFRP-reinforced concrete slabs with a 60 mm cover to ensure safety after a 2 h fire. 

Sawpan [17] evaluated the durability of pultruded GFRP reinforcement bars conditioned in seawater and dry environments at 23, 55, and 75 °C for up to 20 months. The transverse shear strength (TSS) and SBSS measurements indicated that dry conditioning at elevated temperatures increased TSS by 5.9% and 7.6% at 55 and 75 °C, respectively. In contrast, seawater exposure at high temperatures reduced the TSS, with 91.7% retention at 75 °C over 20 months. The SBSS demonstrated slight increases under moderate conditions but decreased by 4.5% under seawater exposure at 75 °C, with the degradation attributed to the weakening of the fiber–matrix interface due to plasticization in seawater. 

D’Antino et al. [18] studied the durability of thermoset (6, 20, and 32 mm diameters) and thermoplastic (6, 12, and 25 mm diameters) GFRP bars in an alkaline solution with pH 13 at 60 °C for up to 1000 h using tensile and short-beam shear tests. The tensile strength and elastic modulus of the thermoset bars decreased by 13.5% and 6.1%, respectively, whereas those of the thermoplastic bars exhibited maximum reductions of 12.4% and 8.3%, respectively. The SBSS was minimally affected, except for the bars with the smallest diameters, which showed significant reductions. 

Machine learning (ML) algorithms have been used to analyze complex data efficiently, reveal hidden patterns, and make accurate predictions without requiring explicit governing equations—especially beneficial for addressing complex, non-linear problems that traditional methods often struggle to adequately address [19,20,21]. An artificial neural network (ANN) is a computational model inspired by the structure and functions of the human brain. ANNs consist of interconnected layers of nodes (or “neurons”) organized into an input layer, one or several hidden layers, and an output layer. ANNs are designed to learn complex data patterns, making them powerful for various applications, such as pattern recognition, natural language processing, and prediction modeling [22,23]. While several studies have reported applying ML techniques for predicting concrete strength with reasonable accuracy [19,24,25,26,27,28], applications [9] of ML models in the SBSS prediction of GFRP bars remain limited.

In this study, the reductions in the SBSS of two new-generation GFRP bars, namely sand-coated (SG) and ribbed (RG) bars, were evaluated after conditioning in four environments—alkaline, a combination of alkaline and salt, acidic, and water—and under two temperature conditions—ambient laboratory and elevated temperatures—for 3, 6, and 12 months. This study aims to investigate the performance of new-generation GFRP bars subjected to harsh conditions and compare the results with the SBSS retention of three types of older-generation GFRP bars exposed to similar conditions. A large amount of experimental data from this research were analyzed to develop multiple linear regression (MLR) and ANN models using MATLAB (R2023b) to create and compare predictive models of the SBSS.

## 2. Methodology

### 2.1. Materials

#### GFRP Bars

**Figure 1:**
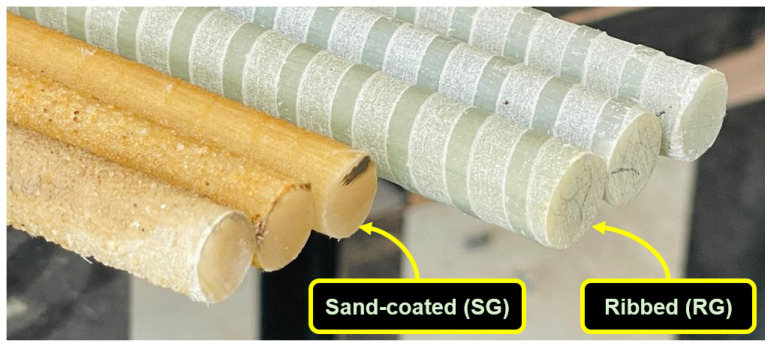
GFRP bar types used in this study.

**Figure 2:**
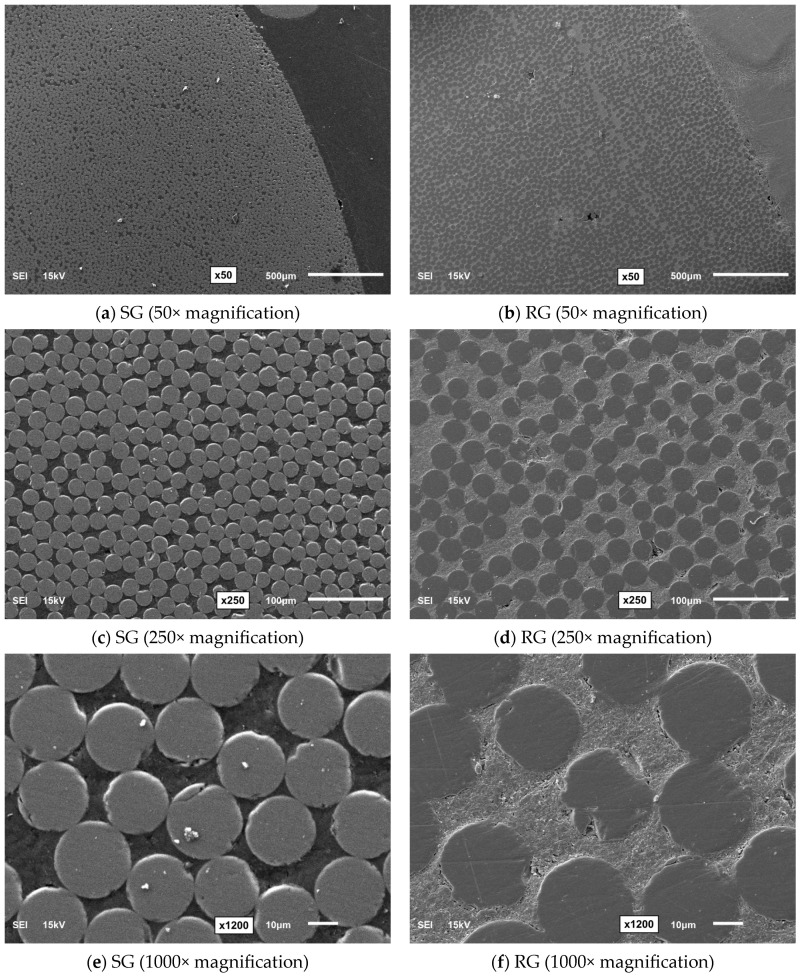
SEM micrographs of the GFRP bars (near the circumference).

In this study, the durability performance of SBSS was investigated for two types of commercially available new-generation GFRP bars—sand-coated (SG) and ribbed (RG)—produced using the pultrusion process, as shown in Figure 1. The SG bar (13.02 mm diameter) has a textured surface with uniformly distributed sand particles, whereas the RG bar (13.71 mm diameter) has evenly spaced spiral ridges along its length. The detailed technical properties of the GFRP bars are listed in Table 1. The scanning electron microscope (SEM) images of the bars magnified at 50×, 250×, and 1000× are presented in Figure 2. The enhanced durability of new-generation GFRP bars over older generations is primarily due to advancements in the resin composition, manufacturing processes, and quality of the fibers [29]. 

### 2.2. Exposure Conditions

The GFRP bars used in this study were subjected to four types of conditioning solutions. Conditioning solution C1 was prepared as per ACI 440.3R-12 [5] by mixing 118.5 g of calcium hydroxide, 0.9 g of sodium hydroxide, and 4.2 g of potassium hydroxide in 1 L of deionized water to achieve a pH value in the range of 12.6–13. Solution C1 simulates the highly alkaline environment of a concrete pore solution with a pH of approximately 13.0 [5,32]. Solution C2, prepared by introducing 3% NaCl into solution C1, simulates the seawater environment. Solution C3, prepared using 0.6% acetic acid [33], helps achieve a pH of 3.1. Solution C4 used pH-neutral tap water to observe the performance of the GFRP bars in a highly humid environment. The effect of temperature on the performance of the samples was evaluated by exposing the samples to approximately 20 °C, representing a controlled indoor laboratory condition, while a high-temperature condition was simulated by placing samples in a 60 °C environment [10,17,33,34]. 

**Figure 3:**
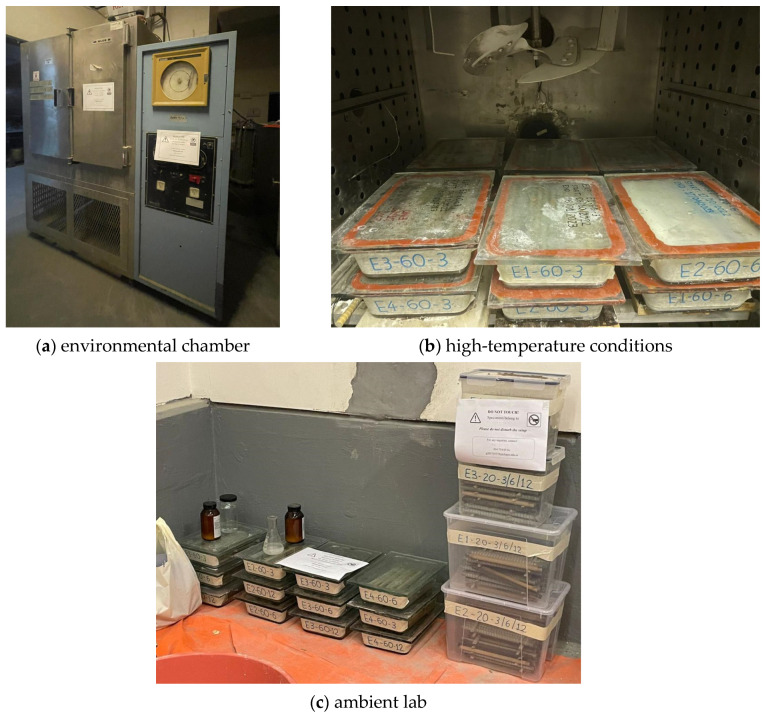
Exposure conditions used in this study.

GFRP bar specimens were cut into lengths of 300 mm, end-coated with epoxy resin, and placed in containers. The containers were then filled with one of the conditioning solutions—C1, C2, C3, or C4—and sealed with silicone to inhibit moisture loss. The containers were then placed in the environmental chamber shown in Figure 3a for high-temperature exposure (Figure 3b) or under standard laboratory conditions (Figure 3c).

### 2.3. Short-Beam Shear Test

**Figure 4:**
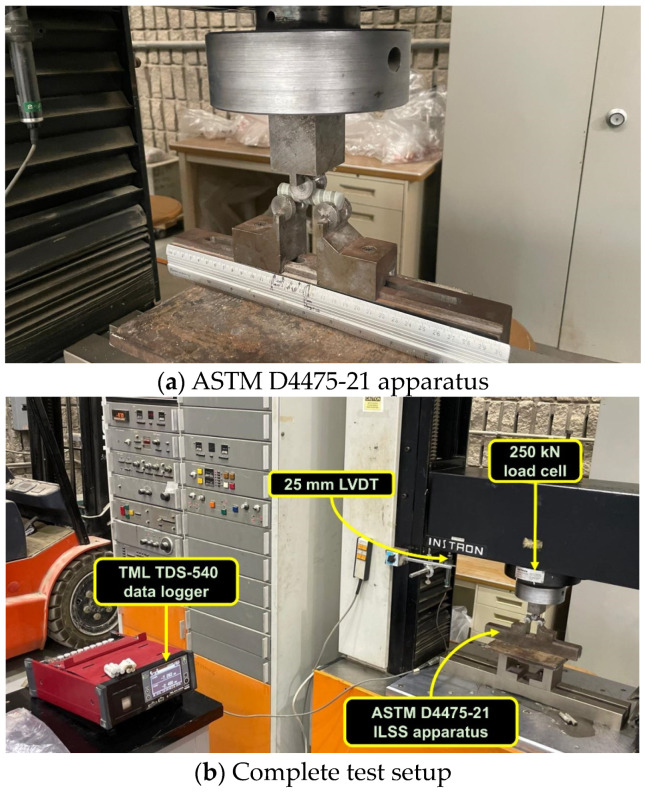
SBSS testing as per ASTM D4475-21 [8].

The SBSS of the GFRP bar specimens was determined following ASTM D4475-21 [8] using an in-house fabricated apparatus, as shown in Figure 4a, with specially fabricated anvils for 13 mm diameter bars. The test specimens were prepared by cutting the GFRP bars to a length four times the diameter of the bar and positioning them on two support anvils, as illustrated in Figure 4a. Loading was applied through the upper anvil to create a horizontal shear plane. The SBSS of the GFRP bars was calculated using the formula provided in ASTM D4475-21 [8], which is referred to as Equation (1).

(1) $$S=0.849\frac{P}{d^{2}}$$

where *S* is the strength of the short beam (MPa), *P* is the failure load (N), and *d* represents the specimen diameter (mm). In this study, displacement-controlled loads were applied at a rate of 1 mm/min. A 250 kN-capacity load cell and a linear variable differential transducer (LVDT) were simultaneously recorded in a data logger, as shown in Figure 4b.

### 2.4. Test Matrix

**Figure 5:**
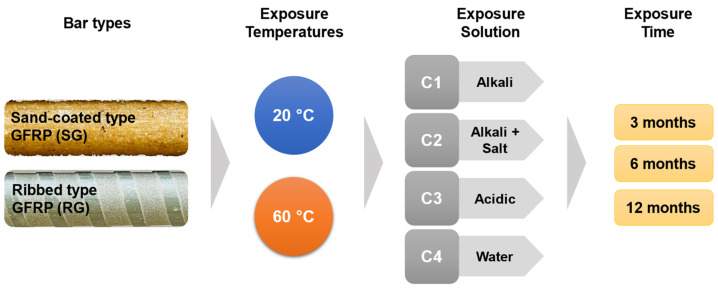
Test matrix (graphical form).

An experimental plan involving 250 GFRP bar specimens to be tested for SBSS (Figure 5) was devised to evaluate every possible exposure combination discussed previously. For each combination, the SBSS was assessed using five replicates, adhering to the ASTM D4475-21 guidelines [8]. Unconditioned control specimens were also tested to establish baseline SBSS values. A summary of the test matrices, including the names of each exposure combination, is presented in Table 2.

### 2.5. Strength Prediction Models

Linear regression is a statistical method that models the relationship between a dependent variable (response) and one or more independent variables (predictors) by fitting a linear equation to the observed data [33,35,36]. The general form of linear regression is expressed in Equation (2), where y is the dependent variable; $x_{1},$ $x_{2},\;\ldots ,\;x_{n}$ are the independent variables; $\beta_{1},$ $\beta_{2},\;\ldots ,\;\beta_{n}$ are the fitting parameters; and ϵ is the error term.

(2) $$y=\beta_{0}+\beta_{1}x_{1}+\beta_{2}x_{2}+\cdots +\beta_{n}x_{n}+\epsilon$$

Linear regression is valuable owing to its simplicity in interpreting variable relationships, making it useful in fields such as economics and engineering [37]. However, linear regression assumes a linear relationship, possibly making it unsuitable for complex data, sensitive to outliers, and susceptible to multicollinearity, thus limiting its performance with non-linear or high-dimensional data.

**Figure 6:**
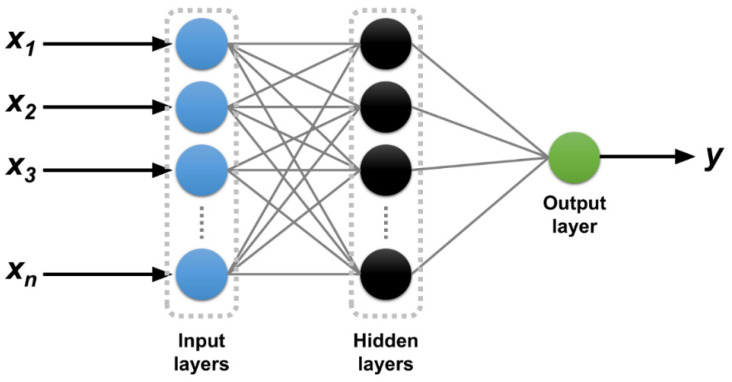
ANN architecture.

ANNs, computational models inspired by the structure of the human brain by mimicking its interconnected neurons, are primarily used in applications involving ML and artificial intelligence (AI) [21,38]. ANNs typically consist of interconnected layers of units (known as “neurons”), which collaboratively process data, recognize patterns, make decisions, and generate predictions. As shown in Figure 6, a standard ANN architecture includes an input layer, one or more hidden layers, and an output layer. Each neuron in a given layer connects to the neurons in the next layer [39]. Each connection carries a weight that adapts as the network learns from data.

Modeling in an ANN begins with the training phase, where a portion of the input and output data are used to help the model learn patterns and trends from the input data. This step is followed by the validation phase in which the performance of the developed model is assessed on a separate subset of the data to refine and prevent overfitting [40]. In the final testing phase, a separate test subset is used to objectively evaluate the accuracy of the fully trained model. For this study, the neural network toolbox in MATLAB (“*nftool*”) was employed to develop a feed-forward back-propagation model using the Bayesian regularization algorithm. In this study, the input dataset was divided into 70%, 15%, and 15% subsets for training, validation, and testing, respectively.

## 3. Results and Discussion

### 3.1. SBSS–Displacement Response

#### 3.1.1. Control Specimens

**Figure 7:**
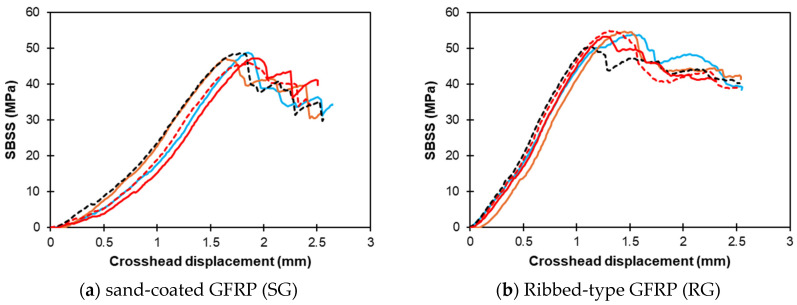
SBSS–crosshead displacement response of control RG and SG bars. Note: multiple lines indicate replicates.

The load–displacement curves of typical control specimens of sand-coated SG and ribbed-type RG GFRP bars are plotted in Figure 7a,b, respectively. The sand-coated SG bars exhibit an average peak SBSS of 47.6 ± 1.2 MPa. The SBSS–displacement curves in the SG bars show an initial sharp rise, representing elastic deformation, followed by a peak between 40 and 50 MPa, before gradually declining with fluctuations, likely due to microcracking. The ribbed-type RG bars exhibit a mean SBSS of 53.6 ± 1.7 MPa. The SBSS–displacement response curves initially showed a sharp increase in strength with displacement, indicating elastic deformation, followed by a peak between 50 and 60 MPa. After the peak, the SBSS decreased with displacement.

#### 3.1.2. Conditioned Bars

**Figure 8:**
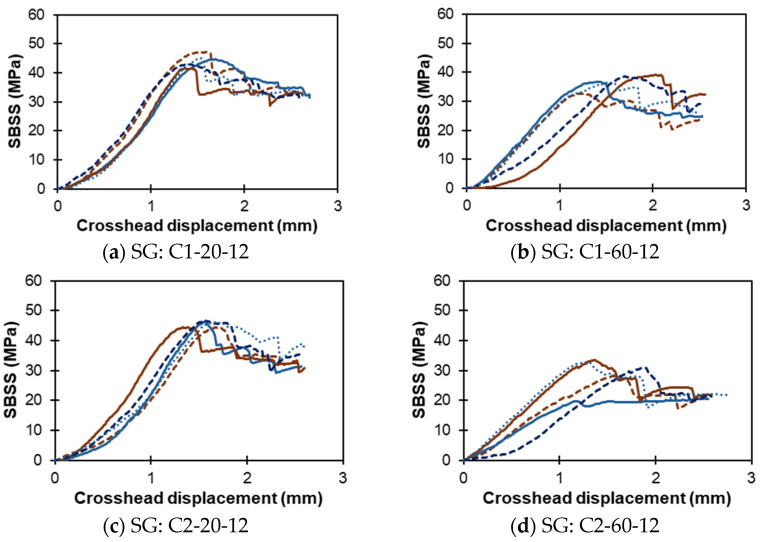

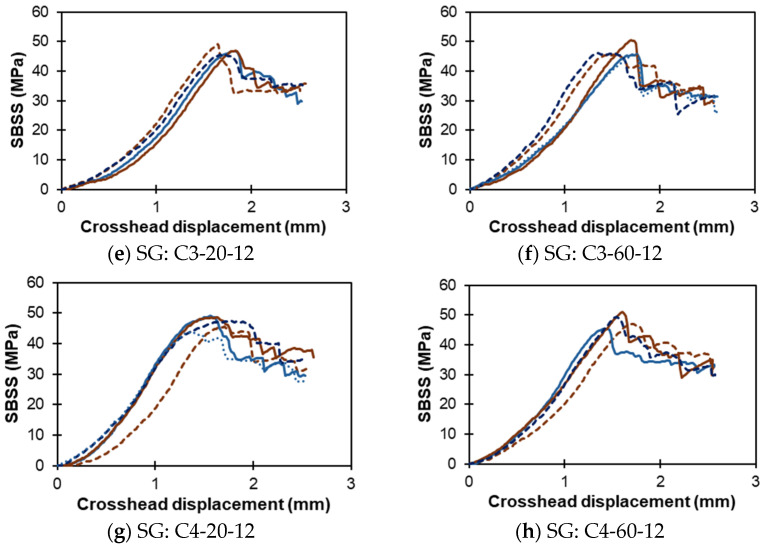
Sand-coated SG bars: SBSS–displacement curves (notation: exposure solution–temperature–exposure duration). Note: multiple lines indicate replicates.

**Figure 9:**
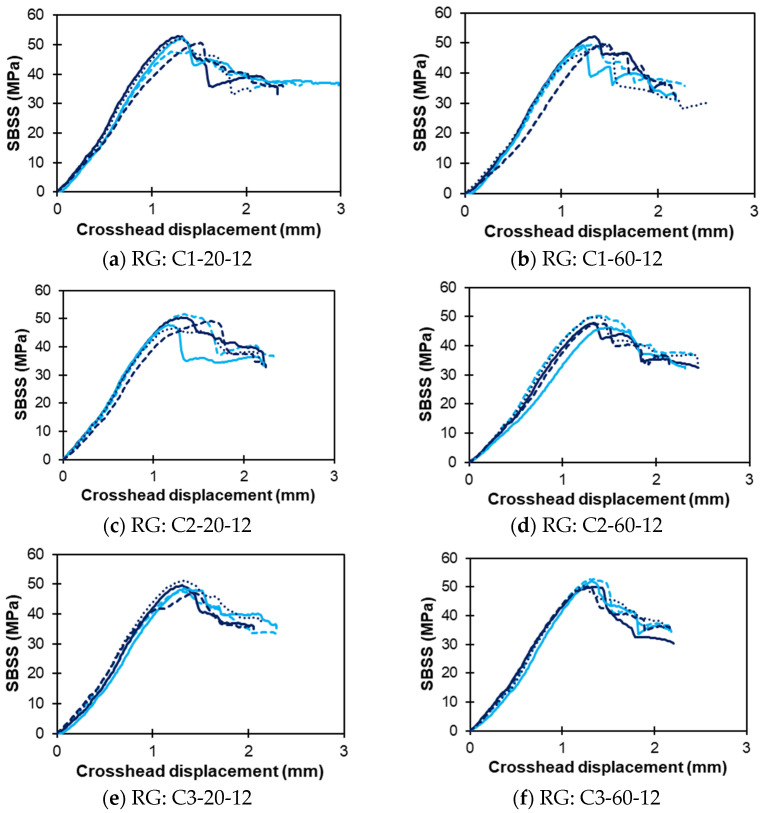

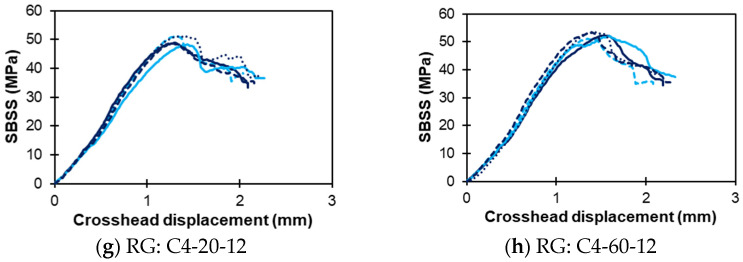
Ribbed-type RG bars: SBSS–displacement curves (notation: conditioning solution–temperature–duration). Note: multiple lines indicate replicates.

Figure 8a–h show the SBSS displacement response of the sand-coated SG bars exposed to solutions C1, C2, C3, and C4 under both temperature conditions at 12 months. Similarly, Figure 9a–h present the SBSS–displacement curves for the ribbed RG bar specimens subjected to the same exposure solutions (C1, C2, C3, and C4) at high and low temperatures after 12 months of conditioning.

**Figure 10:**
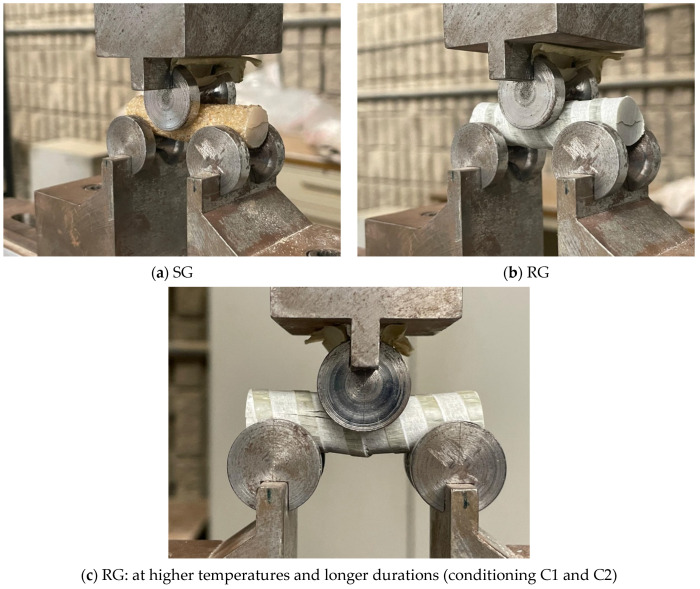
Failure modes.

All of the unconditioned specimens, along with those conditioned for up to six months, failed horizontally along the length of the specimen, initiating at the center and extending toward either the right or left end, as shown in Figure 10a,b. In contrast, the specimens conditioned for 12 months displayed a flexural failure mode, with cracks beginning in the area directly opposite the point of load application (Figure 10c). The observed transition in failure modes highlights the effect of harsh conditioning on the fracture behavior of GFRP bars. The flexural failure observed in specimens conditioned for 12 months indicates a reduction in the SBSS due to prolonged exposure, which leads to a shift in stress distribution and crack initiation. The SEM fractography of the regions near the exposed surface of the GFRP bars is presented later in Section 4 of this paper to provide further insights into the microstructural changes contributing to these fracture behaviors.

### 3.2. Effect of Conditioning on SBSS

**Figure 11:**
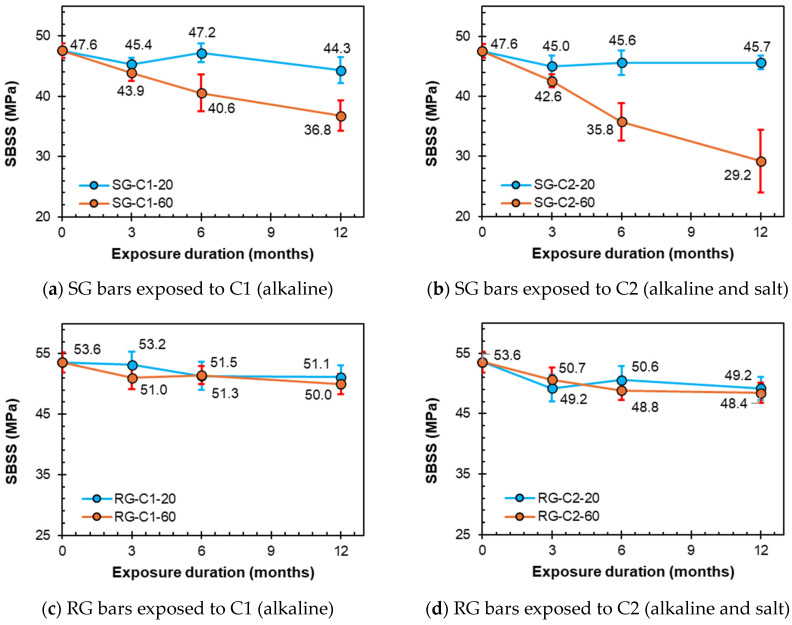
SBSS degradation due to C1 (alkaline) and C2 (alkaline and salt) conditionings.

The effects of the different exposure conditions on the SBSS retention of the GFRP bars in this study are discussed in this section. Figure 11a,b show the progression of sand-coated SG bars across three exposure durations (3, 6, and 12 months) under C1 (alkaline) and C2 (alkaline and salt) conditions, respectively. Under ambient laboratory conditions (20 °C), the bars demonstrated good resilience to the alkaline solutions, with reductions of 6.9% and 4.1% for C1 and C2, respectively. However, the SG bars exhibited significant SBSS reductions of 22.7% and 38.5% for C1 and C2, respectively. The strength reduction remained steady throughout the exposure period.

The ribbed RG bars exhibited reductions of 4.5% and 8.2% in C1 and C2, respectively, after 12 months (Figure 11c,d). However, RG exhibited better SBSS retention compared to SG at high-temperature conditions at 60 °C. In C1 and C2 at 12 months, the RG showed only 6.6% and 9.6% reductions in the SBSS, respectively.

**Figure 12:**
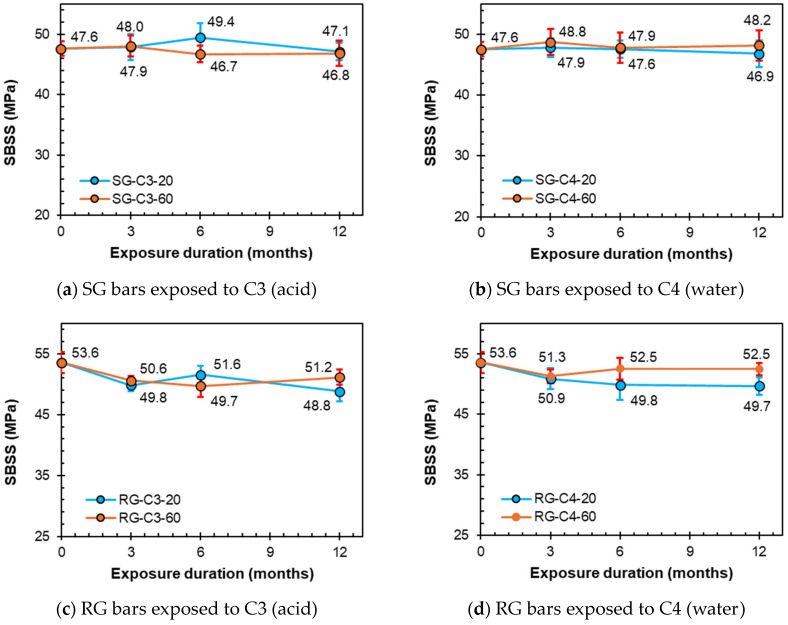
SBSS degradation due to C3 (acidic) and C4 (water) conditionings.

The effects of the conditioning solutions C3 (acidic) and C4 (water) on the SG and RG bars are shown in Figure 12. The evolution of the SBSS of the sand-coated SG bars for various conditioning durations (3, 6, and 12 months) in C3 and C4 is shown in Figure 12a,b, respectively. Under laboratory conditions at 12 months, the bars demonstrated low SBSS reductions of 1.0% and 1.5% at C3 and C4, respectively. Unlike C1 and C2, C3 and C4 caused a minimal SBSS reduction of 1.7% in C3 and a mild increase of 1.3% in C4 after 12 months. 

The effects of C3 and C4 conditioning solutions on the SBSS retention of the ribbed RG bars for various conditioning durations (3, 6, and 12 months) are shown in Figure 12c,d, respectively. Under laboratory conditions, the bars exhibited SBSS reductions of 8.8% and 7.3% for C3 and C4, respectively. Conditioning solutions C3 and C4 caused reductions of 4.5% and 2.0%, respectively, after 12 months of high-temperature exposure.

### 3.3. Comparison with Older-Generation Bars

#### 3.3.1. Reference Work

**Figure 13:**
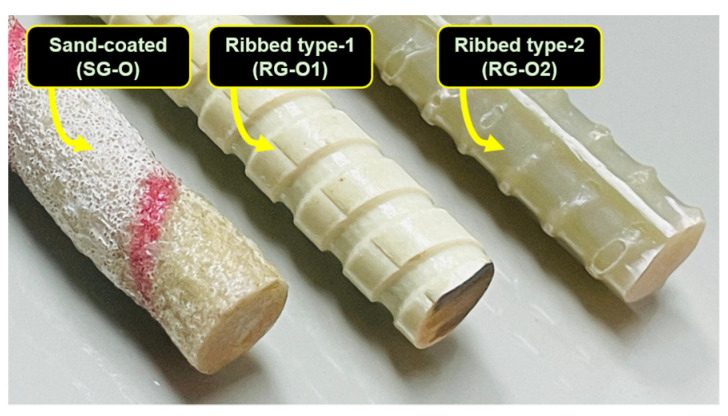
Surface features of older-generation GFRP bars.

This study investigated the effects of various accelerated aging conditions on two types of new-generation GFRP bars. Older-generation bars exposed to similar conditions were compared to gain further insights. Accordingly, this study compared the SBSS results with the findings from a previous investigation, which evaluated the SBSS retention of three types of GFRP bars manufactured more than 20 years ago [9,33]. In the research work by Fasil and Al-Zahrani [9], three GFRP bar types, ribbed-type 1 (RG-O1), ribbed-type 2 (RG-O2), and sand-coated (SG-O) GFRP bars, were exposed to four conditions—alkaline, alkaline and salt, acidic, and water—and two temperature regimes—laboratory ambient and high temperature—for up to 12 months. Figure 13 illustrates the surface textures of the SG-O, RG-O1, and RG-O2 bars. The properties of these older-generation GFRP bars, such as their diameter, glass fiber type, matrix type, and transverse shear strength, are summarized in Table 3. 

#### 3.3.2. Effect of Conditioning

**Figure 14:**
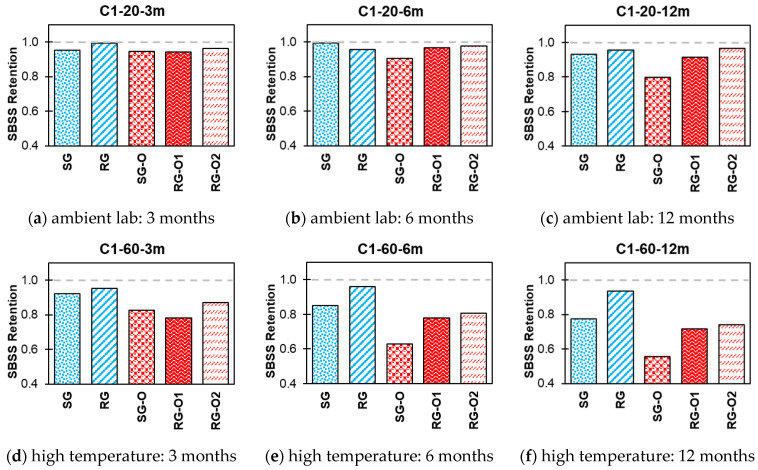
Effect of C1 (alkaline) SBSS retention.

**Figure 15:**
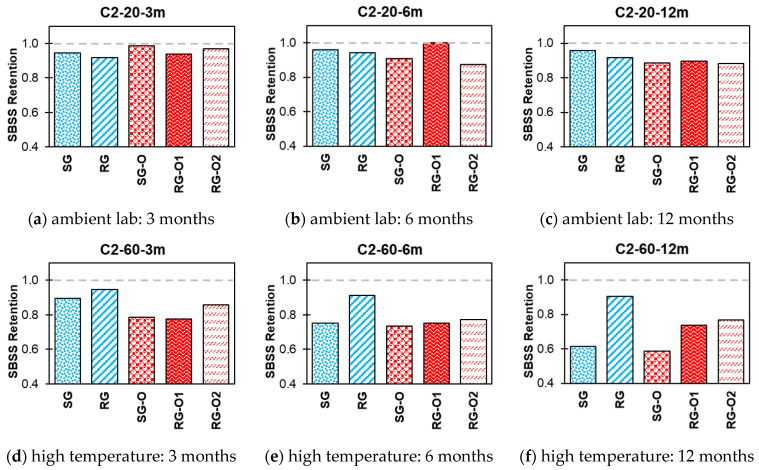
Effect of C2 (alkaline and salt) SBSS retention.

**Figure 16:**
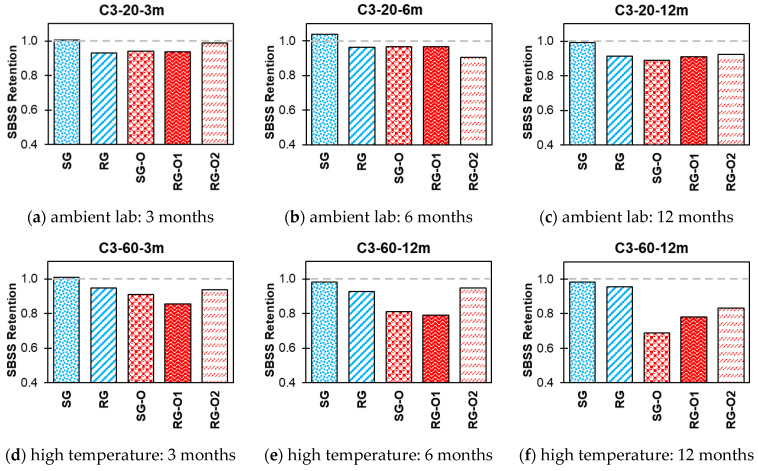
Effect of C3 (acid) SBSS retention.

**Figure 17:**
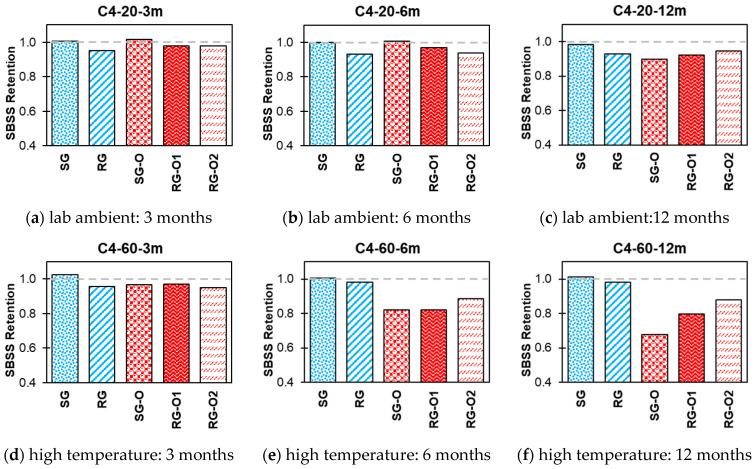
Effect of C4 (water) SBSS retention.

The impact of various exposure conditions used in this study, i.e., alkaline, alkaline and salt, acid, and water, on the SBSS retention of both new-generation bars (SG and RG) and older-generation bars (SG-O, RG-O1, and RG-O2) are shown in Figure 14, Figure 15, Figure 16 and Figure 17. 

In C1, at 20 °C, all bar types exhibited relatively high retention over time (Figure 14). The SG and RG bars retained 93–95% of their initial SBSS values after 12 months, indicating strong resilience under ambient conditions. The older-generation bars (SG-O, RG-O1, and RG-O2) also exhibited good retention values in the range of 80–96% after 12 months. At a higher temperature of 60 °C, a noticeable decline in SBSS retention was observed across all bar types over time. The new-generation SG and RG bars retained 77% and 93% of their initial SBSS values, respectively, after 12 months, whereas the older-generation bars exhibited greater reductions, with SG-O retaining 56%, RG-O1 retaining 71%, and RG-O2 retaining 74% after 12 months. 

In C2 at 20 °C (Figure 15), the SG and RG bars retained approximately 96% and 92%, respectively, of their initial SBSS values after 12 months. The older-generation bars (SG-O, RG-O1, and RG-O2) also exhibited good retention, with values between 88% and 90% over 12 months. However, at 60 °C, notable reductions in SBSS retention were observed across all bars with increasing exposure durations. After 12 months, the SG and RG bars retained 61% and 90% of their initial SBSS values, respectively. The older-generation bars experienced notable reductions, with SG-O at 59%, RG-O1 at 74%, and RG-O2 at 77% retention after 12 months. 

In C3 at 20 °C (Figure 16), the SG bar showed a slight increase, with values exceeding 100% (1.01 and 1.04) after 3 and 6 months and stabilizing at 99% after 12 months. The RG, SG-O, RG-O1, and RG-O2 bars also retained 89–92% of their SBSS values after 12 months. At 60 °C, SBSS retention declined over time, particularly for the older-generation bars. By 12 months, while the SG and RG bars retained 98% and 96% of their initial SBSS values, respectively, the older-generation bars showed greater reductions, with SG-O at 69%, RG-O1 at 78%, and RG-O2 at 83% after 12 months of conditioning. 

In conditioning solution C4 at 20 °C, most bars maintained high SBSS retentions, with values of 98% and 93% for the SG and RG bars (Figure 17). The SG-O, RG-O1, and RG-O2 bars showed retentions of 90%, 92%, and 95%, respectively, after 12 months. At 60 °C exposure after 12 months, the SG and RG bars retained their initial SBSS values. By contrast, the older bars experienced more significant reductions, with SG-O retaining 68%, RG-O1 retaining 80%, and RG-O2 retaining 88% after 12 months. These results suggest that the new-generation bars, particularly SG and RG, exhibit better resistance to all water exposure conditions at high temperatures than their older counterparts.

The new-generation GFRP bars (SG and RG) generally demonstrated superior SBSS retention values compared with the older-generation bars (SG-O, RG-O1, and RG-O2) across various exposure conditions and temperatures. While both the new and old bars perform well at ambient temperatures, the new bars, especially RG, exhibit significantly better durability and resistance to strength degradation at elevated temperatures (60 °C).

## 4. Effect of Conditioning on Morphology of GFRP Bars

**Figure 18:**
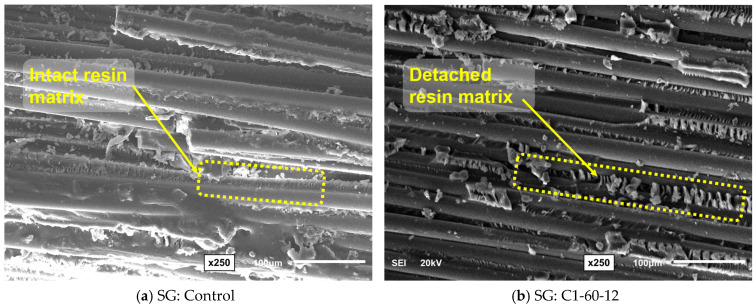
SEM fractography (250× magnification): effect of conditioning C1 on SG bar at 12 months of exposure at 60 °C.

**Figure 19:**
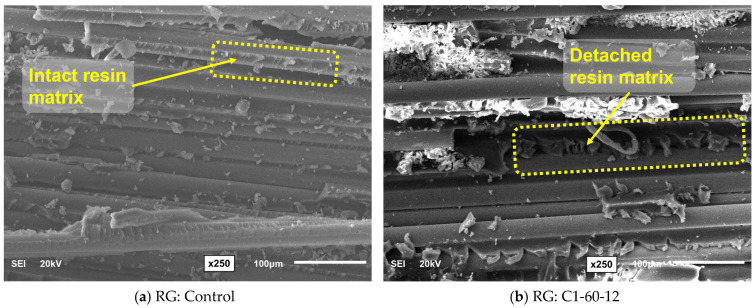
SEM fractography (250× magnification): effect of conditioning C1 on RG bar at 12 months of exposure at 60 °C.

The microstructural analyses of the two types of GFRP bar specimens were conducted using SEM to evaluate the effect of prolonged alkaline exposure on the material’s fracture surface. Samples were obtained from the fracture surfaces following the SBSS testing to validate the cause of degradation in their mechanical performance. Figure 18 and Figure 19 compare the SEM fractography to assess the effect of C1 on SG and RG bars, respectively. The control specimens of SG and RG, shown in Figure 18a and Figure 19a, respectively, exhibited relatively smooth fracture surfaces with minimal resin detachment and intact fiber-matrix adhesion. However, the SG and RG specimens that were exposed to the alkaline solution (C1) at 60 °C for one year, as shown in Figure 18b and Figure 19b, respectively, presented significant surface degradation, characterized by resin detachment and fiber exposure.

The damage observed in the resin of the exposed specimens compromises the structural role of the resin matrix and the fiber-matrix interface, which contributes to the significant reduction in the SBSS of GFRP bars after prolonged conditioning in alkaline environment C1.

## 5. Short-Beam Shear Strength (SBSS) Prediction Models

### 5.1. Dataset

A dataset containing the SBSS test results for two types of new-generation GFRP bars (RG and SG) and three types of older-generation GFRP bars (RG-O1, RG-O2, and SG-O) under various exposure conditions was used to develop the linear regression and ANN models. Predictor variables included exposure types (C1, C2, C3, and C4), temperatures (20 °C and 60 °C), and bar types (SG, RG, SG-O, RG-O1, and RG-O2, SG-O) with respective diameters of 13.02, 13.71, 13.22, 13.04, and 13.02 mm. The response or target variable, the SBSS, ranged from 25.6 to 56.5 MPa. Categorical data such as exposure and bar type were represented using dummy variables (0 and 1). The coefficient of determination (*R*^2^) was used to evaluate the performance of both models, with higher *R*^2^ values (closer to 1) indicating better accuracy.

### 5.2. Linear Regression 

**Figure 20:**
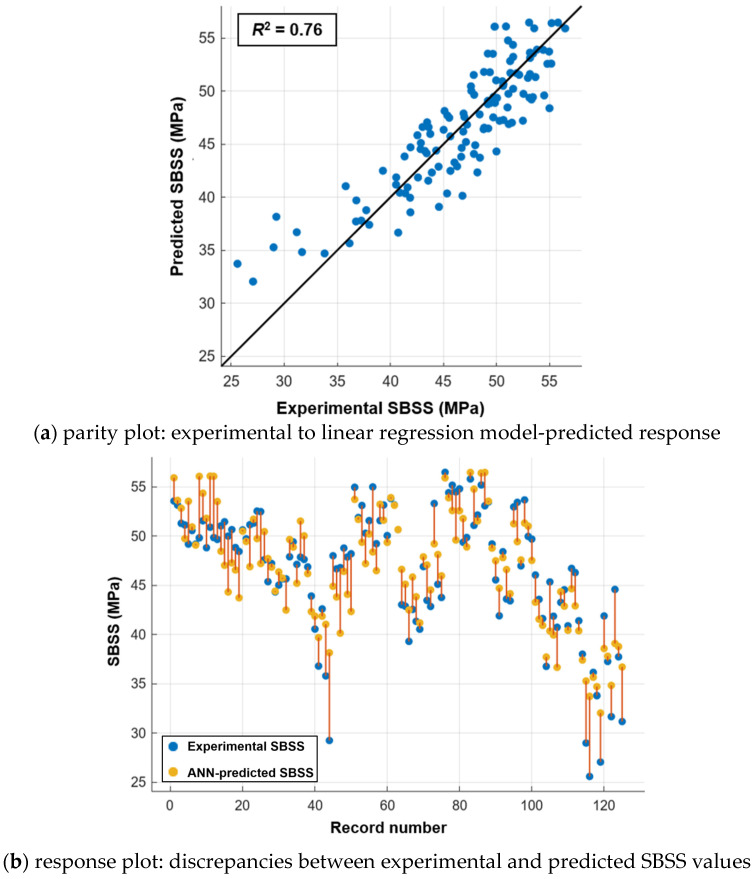
Linear regression model.

The parity plot in Figure 20a compares the experimental and predicted SBSS values obtained using the linear regression model. The model overestimates the SBSS values at lower strengths, particularly for values up to 40 MPa. The linear regression model achieved an *R*^2^ value of 0.76, indicating a relatively low reliability. The response plot in Figure 20b shows the variations between the experimental and predicted SBSS values for every input used in the model, highlighting discrepancies in the predictions.

### 5.3. ANN Model

**Figure 21:**
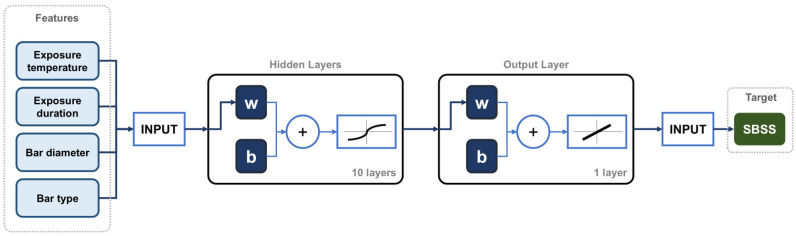
Structure of ANN used in MATLAB (w = weight; b = bias).

**Figure 22:**
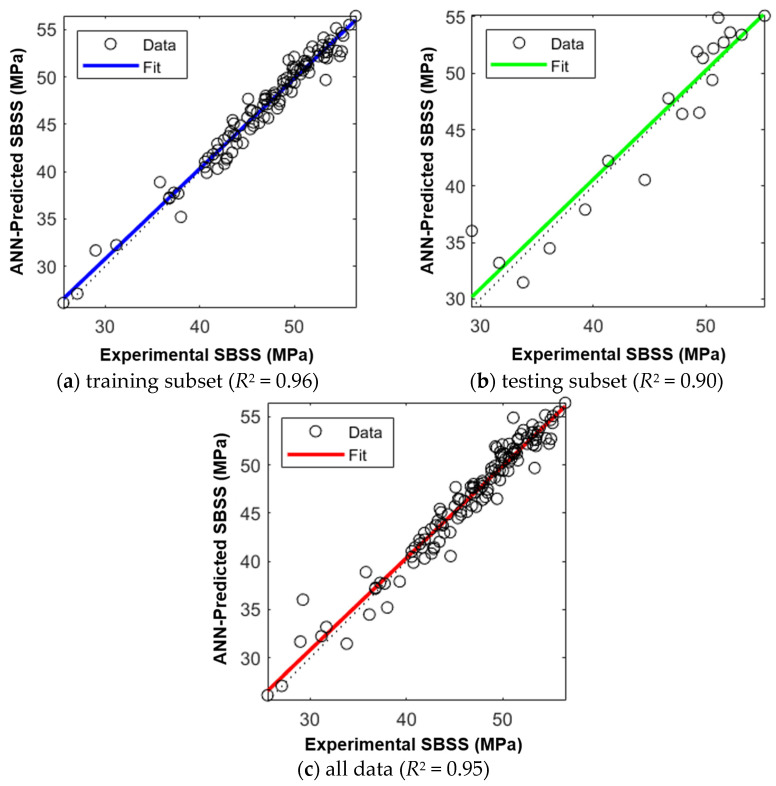
Parity plots—experimental vs. ANN prediction of SBSS.

**Figure 23:**
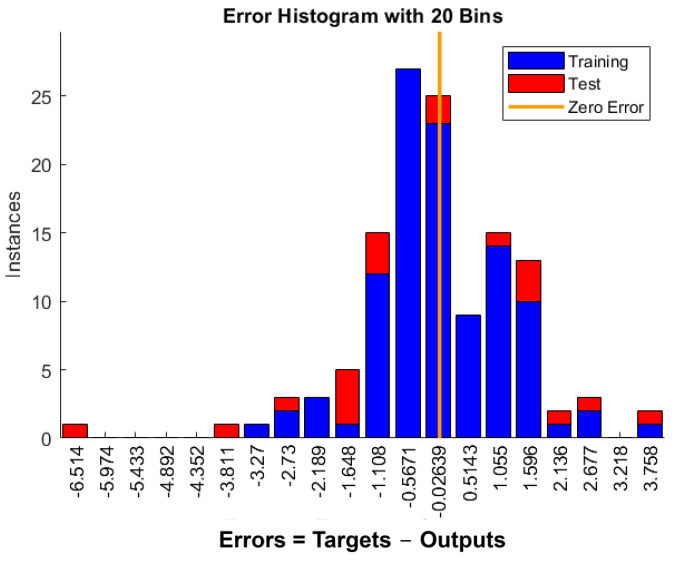
A frequency histogram of the error distribution with 20 bins.

The ANN developed in this study, illustrated in Figure 21, is a two-layer feed-forward network with ten hidden neurons using a sigmoidal activation function and a single output layer with a linear transfer function. The ANN model’s performance, assessed through parity plots in Figure 22, shows an effective prediction of the SBSS for GFRP bars, with *R*^2^ values of 0.96, 0.90, and 0.95 for the training (Figure 22a), testing (Figure 22b), and total subsets (Figure 22c), respectively. A frequency histogram with 20 bins, shown in Figure 23, shows the error distribution between the experimental (target) and predicted SBSS values across the training, validation, and testing subsets.

The ANN model demonstrated a significantly higher predictive accuracy (*R*^2^ = 0.95) compared to the linear regression model (*R*^2^ = 0.76). This enhanced accuracy is crucial for practical applications such as predicting the long-term durability of GFRP bars under varying environmental conditions, optimizing material formulations, and informing performance-based design codes. Given that the training process for the ANN is a one-time computational effort, its ability to provide quick and accurate predictions for a wide range of exposure scenarios makes it a valuable and efficient tool. The enhanced performance of the ANN model over the linear regression model is attributed to its capability to capture and model non-linear relationships between the predictor and target variables, thus providing more accurate predictions in complex datasets.

## 6. Conclusions

In this study, the durability performance of GFRP bars with sand-coated (SG) and ribbed (RG) surface textures in four conditioning solutions (C1 (alkaline), C2 (alkaline and salt), C3 (acid), and C4 (water)), two temperature levels (ambient laboratory temperature and high temperature), and three conditioning durations (3, 6, and 12 months) was investigated. The following conclusions were drawn:

- The SBSS retention results show that both the sand-coated SG and ribbed RG GFRP bars demonstrated strong resilience under ambient laboratory conditions with no significant reduction in strength until 12 months. However, the SBSS noticeably declined under high-temperature conditions, with the SG bars showing a 22.7% reduction and the RG bars showing a mild 6.6% reduction in the alkaline solution (C1). In the alkaline and salt solutions (C2), the SG bars exhibited a 38.6% decrease, whereas the RG bars showed a slight decrease of 9.6%.
- Acidic (C3) and water (C4) conditions caused a minimal reduction in strength irrespective of the exposure duration and temperature.
- SEM fractography revealed significant degradation in the resin matrix and fiber–matrix interface in the GFRP bars exposed to prolonged alkaline conditioning (C1) at 60 °C, including resin detachment and fiber exposure, which contributed to the reduction in the SBSS.
- Generational advancements in GFRP durability were assessed by comparing three types of older-generation bars—one sand-coated (SG-O) bar and two ribbed types (RG-O1 and RG-O2). The new-generation bars outperformed the older-generation GFRP bars in all conditioning scenarios.
- Prediction models using linear regression and ANN were developed and compared. The ANN model demonstrated superior accuracy with an *R*^2^ value of 0.95 compared to 0.76 for the linear regression model, reflecting the ability of the ANN to account for the non-linear behavior of strength retention in GFRP bars under various exposure conditions. By integrating diverse datasets and simulating complex exposure scenarios, ANN can enhance our understanding of the SBSS behavior in fiber-reinforced composites, optimize material design, and assist in durability assessments.

## Figures and Tables

**Table 1:** GFRP bars’ properties.

Bar Type	Sand-Coated Type GFRP (SG)	Ribbed Type GFRP (RG)
Diameter from immersion test (mm) [30,31]	13.02 ± 0.083	13.71 ± 0.049
Surface type	Sand-coated	Ribbed
Matrix type *	Vinyl ester resin	Vinyl ester resin
Glass fiber type *	E-glass	E-glass
Ultimate tensile strength (MPa) [30]	1029.6 ± 27.5	956.4 ± 18.5
Ultimate strain (%) [30]	~2.4%	~1.9%
Young’s modulus (GPa) [30]	43.6 ± 3.3	49.5 ± 1.0
Short-beam shear strength (MPa)	47.6 ± 1.2	53.6 ± 1.7
Transverse shear strength (MPa) [30]	185.1 ± 10.2	180.1 ± 3.1
24 h moisture uptake (%) [30]	0.027 ± 0.0007	0.019 ± 0.0007

Note: E-glass = electrical glass; * from manufacturer.

**Table 2:** Test matrix with names.

Conditioning Solution	Conditioning Temperature	Conditioning Duration (Months)	Conditioning ID
C1 (alkaline)	20 °C	3	C1-20-3
		6	C1-20-6
		12	C1-20-12
	60 °C	3	C1-60-3
		6	C1-60-6
		12	C1-60-12
C2 (alkaline and salt)	20 °C	3	C2-20-3
		6	C2-20-6
		12	C2-20-12
	60 °C	3	C2-60-3
		6	C2-60-6
		12	C2-60-12
C3 (acid)	20 °C	3	C3-20-3
		6	C3-20-6
		12	C3-20-12
	60 °C	3	C3-60-3
		6	C3-60-6
		12	C3-60-12
C4 (water)	20 °C	3	C4-20-3
		6	C4-20-6
		12	C4-20-12
	60 °C	3	C4-60-3
		6	C4-60-6
		12	C4-60-12

**Table 3:** Properties of older-generation GFRP bars [9,33].

Bar Type	Sand-Coated Type (SG-O)	Ribbed Type-1 (RG-O1)	Ribbed Type-2 (RG-O2)
Surface texture	Sand-coated	Ribbed	Ribbed
Diameter (mm) (immersion test)	13.22	13.04	13.02
Glass fiber type	E-CR-glass	E-glass	E-glass
Matrix type	Vinyl ester resin	Vinyl ester resin	Urethane-modified vinyl ester resin
24 h moisture uptake (%)	0.092	0.046	0.055
Short-beam shear strength (MPa) [9]	46.1	55.0	56.5
Transverse shear strength (MPa) [33]	179.0	180.3	200.6

Note: E-glass = electrical glass.

## Data Availability

The original contributions presented in this study are included in the article; further inquiries can be directed to the author.
